# Acute obstructive appendicitis in a child caused by pinworms treated with endoscopic direct appendicitis therapy

**DOI:** 10.1055/a-2387-3979

**Published:** 2024-09-04

**Authors:** Jun Cai, Yanli Wang, Silin Huang, Suhuan Liao, Yi Tan, Defeng Cai, Jin Huang

**Affiliations:** 1 Department of Gastroenterology, South China Hospital, Medical School, Shenzhen University, Shenzhen, China; 2 Department of Paediatrics, South China Hospital, Medical School, Shenzhen University, Shenzhen, China; 3 Department of Anesthesiology, South China Hospital, Medical School, Shenzhen University, Shenzhen, China; 4 Department of Clinical Laboratory, South China Hospital, Medical School, Shenzhen University, Shenzhen, China; 5 Medical Department, Shenzhen Exit-Entry Frontier Inspection Station Hospital, Shenzhen, China

A 10-year-old girl presented with a 1-day history of lower right abdominal pain, anorexia, nausea, and vomiting. Ultrasound revealed a tortuous appendix lumen with thick and rough walls ( Fig. 1 **a** ). Computed tomography (CT) scan showed an enlarged appendix with high-density fecaliths and enlarged lymph nodes ( Fig. 1 **b** ), leading to a diagnosis of acute obstructive appendicitis. After obtaining informed consent, we performed endoscopic direct appendicitis therapy (EDAT) using a 9-Fr cholangioscope (EyeMax; Micro-Tech, Nanjing, China) ( Video 1 ), which revealed numerous fecaliths caused by parasites in the appendiceal cavity ( Fig. 2 **a** ). These slightly white fecaliths, containing white-striped parasites, were extracted using a disposable basket ( Fig. 2 **b** ) and flushed with metronidazole and saline solution ( Fig. 2 **c** ). Numerous parasites were evacuated by negative pressure ( Fig. 2 **d** ), leaving the mucosa mildly congested ( Fig. 3 **a** ). The patient's abdominal discomfort rapidly improved. Follow-up CT showed the disappearance of the fecaliths and reduced inflammation ( Fig. 3 **b** ). Laboratory results confirmed the presence of Enterobius vermicularis, or pinworms ( Fig. 4 **a–d** ).

**Fig. 1:**
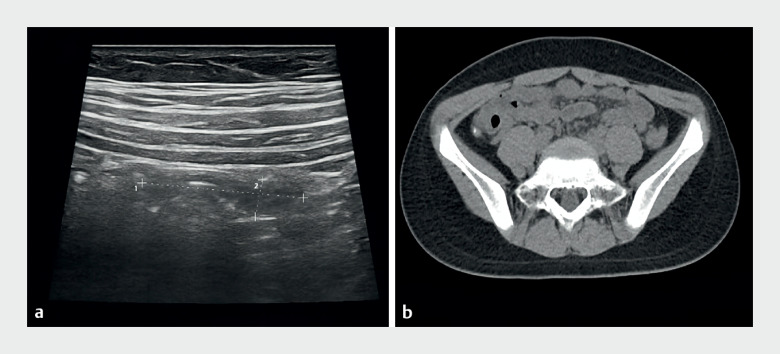
**a** Ultrasound indicated abnormal curved tubular echo with thick and rough walls, suggesting acute appendicitis. **b** Computed tomography (CT) revealed enlarged appendix, fecal stones of high density, and enlarged surrounding lymph nodes.

**Fig. 2:**
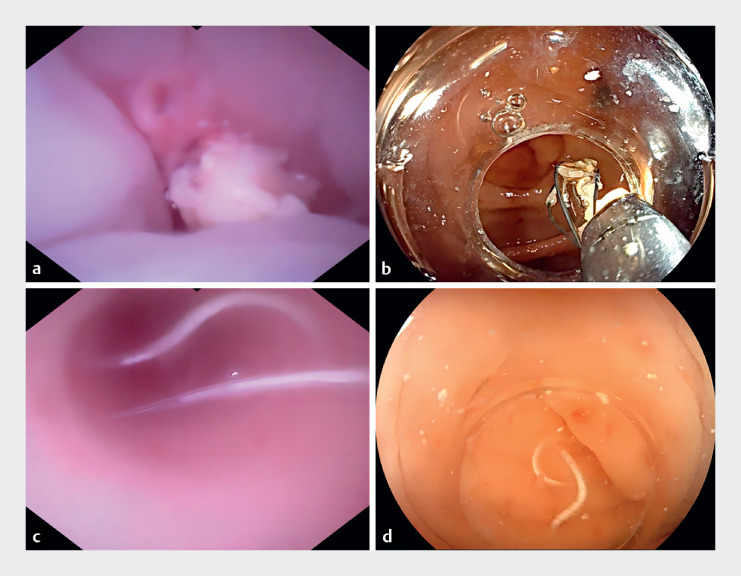
Endoscopic direct appendicitis therapy. **a** Fecaliths caused by parasites in the appendix cavity were slightly white through the cholangioscope. **b** Several fecaliths containing white striped parasites were meticulously extracted under the visual guidance of the cholangioscope. **c** Free live pinworms in the appendix cavity were flushed following repeated lavages with metronidazole and sodium chloride. **d** A large number of free parasites were flushed out into the intestinal cavity; they appear white and slender.

**Fig. 3:**
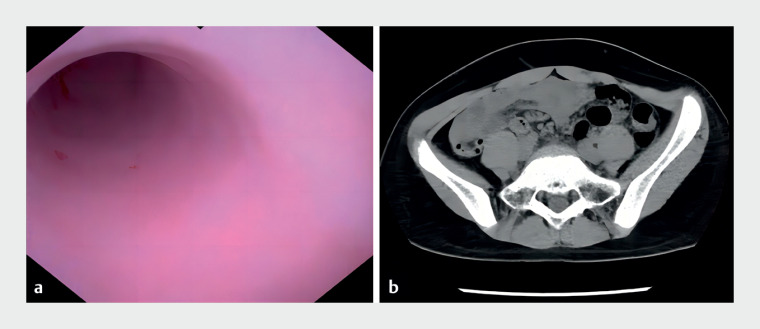
**a** Through the cholangioscope, the appendiceal cavity was observed to be clear and the mucosa mildly congested. **b** Postoperative CT demonstrated disappearance of fecal stones compared to preoperative CT.

**Fig. 4:**
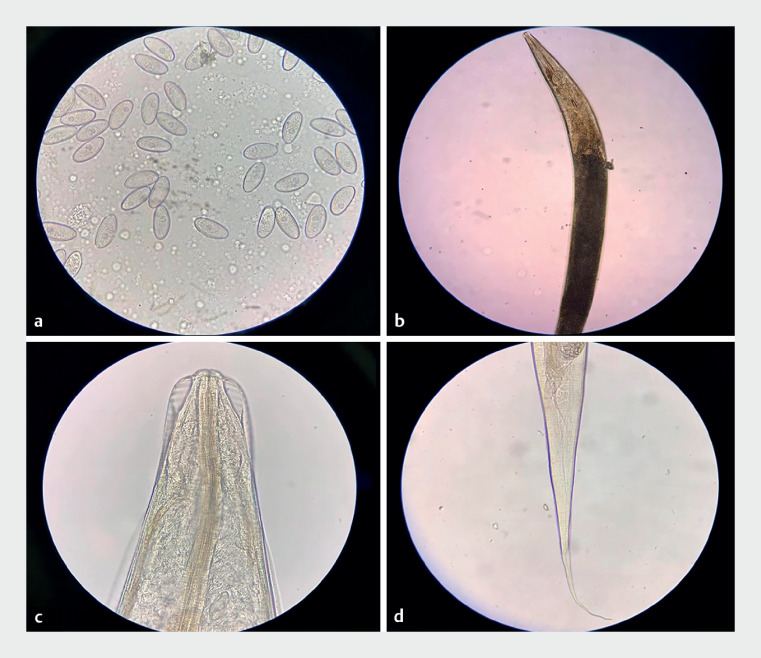
The microscopic observation of E-vermicularis’ eggs in the liquid drawn out by negative pressure in the appendix cavity. **a** E-vermicularis eggs were colorless and transparent with a thick eggshell and no egg cover, resembling grains of rice. **b** The body of E-vermicularis: enlargement. **c** The head of E-vermicularis: the cuticle has horizontal stripes and expands to form the head wing. **d** The tail of E-vermicularis: straight and pointed.

Acute obstructive appendicitis in a child caused by pinworms treated with endoscopic direct appendicitis therapy.Video 1

Enterobius vermicularis accounts for 7% of acute appendicitis cases and is associated with a higher rate of unnecessary appendectomies 1 . Traditionally, the diagnosis depended on surgical and pathological findings 2 3 . This case demonstrates the live morphology of pinworms in the appendix through a cholangioscope, illustrating how pinworms cause appendicitis by promoting the formation of fecaliths and subsequent obstruction. The patient was discharged on the first day after EDAT, highlighting its potential for minimally invasive treatment. This approach offers significant diagnostic advantages over other modalities and emphasizes the importance of technological integration in endoscopic practices.

Endoscopy_UCTN_Code_CCL_1AD_2AZ

## Notes

Endoscopy E-Videos https://eref.thieme.de/e-videos
*E-Videos* is an open access online section of the journal *Endoscopy* , reporting on interesting cases and new techniques in gastroenterological endoscopy. All papers include a high-quality video and are published with a Creative Commons CC-BY license. Endoscopy E-Videos qualify for HINARI discounts and waivers and eligibility is automatically checked during the submission process. We grant 100% waivers to articles whose corresponding authors are based in Group A countries and 50% waivers to those who are based in Group B countries as classified by Research4Life (see: https://www.research4life.org/access/eligibility/ ). This section has its own submission website at https://mc.manuscriptcentral.com/e-videos .
